# A Cohort Study Protocol of Nutritional Status and Clinical Outcomes in Patients With Common Malignancies (NCOM Study)

**DOI:** 10.1002/fsn3.72233

**Published:** 2026-08-24

**Authors:** Hexiang Yang, Yaxin Yu, Haige Cao, Feng Liu, Jiahe Zhou, Yuluyuan Tian, Yilan Li, Xinyue Wang, Yushuo Sun, Xiaoqin Luo

**Affiliations:** ^1^ Department of Nutrition and Food Safety, School of Public Health Xi'an Jiaotong University Xi'an China

**Keywords:** cancer, clinical outcomes, cohort study, malnutrition, nutritional status

## Abstract

Malnutrition is common among patients with cancer and is associated with poorer treatment tolerance, impaired quality of life, and reduced survival. However, evidence regarding the relationship between longitudinal changes in nutritional status and clinical outcomes remains limited. The Nutritional Status and Clinical Outcomes in Patients with Common Malignancies (NCOM) study is an ongoing prospective, multicenter cohort in China. Patients are recruited from 11 hospitals and undergo nutritional assessments conducted within 48 h of admission. Follow‐up is scheduled at 1, 2, 3, 6, and 12 months, followed by annual assessments for an additional 4 years. Baseline nutritional status, assessed using the Modified Patient‐Generated Subjective Global Assessment (mPG‐SGA), is the primary exposure. The primary nutritional and clinical outcomes are longitudinal change in mPG‐SGA and overall survival, respectively. The cohort also captures clinical information, dietary assessment, nutritional support, biomarkers, quality of life, psychosocial factors, physical activity, sleep, nutrition‐related knowledge, attitudes and practices, adverse events, and survival outcomes. Preliminary baseline data from 604 enrolled patients indicated substantial nutritional impairment. According to the mPG‐SGA, 58.61% were classified as having moderate or severe malnutrition, whereas 23.34% were at nutritional risk by the NRS‐2002. These findings support early identification and longitudinal nutritional monitoring in oncology care. Continued follow‐up will enable evaluation of nutritional trajectories and their associations with treatment tolerance, patient‐centered outcomes, and survival.

**Trial Registration:** ClinicalTrials.gov: NCT06219083

## Graphical abstract

The NCOM study is an ongoing multicenter prospective cohort across 11 tertiary hospitals in Xi'an, China, integrating multidimensional assessments of nutritional status and clinical outcomes over 5 years of follow‐up. Preliminary baseline data from 604 participants showed that 58.61% had moderate or severe malnutrition by mPG‐SGA, whereas 23.34% were at nutritional risk by NRS‐2002. These findings support early identification and longitudinal nutritional monitoring in oncology care.

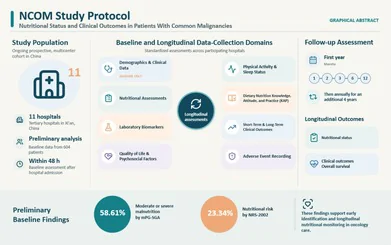

## 1 Introduction

Cancer continues to pose a significant burden on global health. In 2022, an estimated 20 million new cancer cases and 9.7 million cancer‐related deaths occurred worldwide. It is estimated that approximately one in five people will develop cancer during their lifetime (Bray et al. 2024). In China, the burden of cancer remains substantial and is increasingly influenced by population aging, demographic transition, and lifestyle‐related risk factors (Cao et al. 2021; Qiu et al. 2021).

Malnutrition is a frequent and significant problem in oncology clinical practice. Its reported prevalence ranges from 16% to 40% at the time of diagnosis and may rise to 40% to 80% during treatment, depending on age, tumor type, disease stage, and treatment modality (Bossi et al. 2021; Kaegi‐Braun et al. 2021). The burden is particularly severe in cancers that directly affect food intake or gastrointestinal function, including pancreatic, esophageal, gastric, head and neck, and lung cancers (Bossi et al. 2021; Hébuterne et al. 2014).

Nutritional status is closely associated with various clinical outcomes in patients with cancer. Both tumor‐related factors and anticancer treatments can compromise nutritional health by reducing dietary intake, increasing symptom burden, and contributing to metabolic and inflammatory changes (Holdoway et al. 2023; Kipouros et al. 2023; Muscaritoli et al. 2019; Webb et al. 2020). Malnutrition has been associated with poorer treatment tolerance, higher morbidity, impaired quality of life, and reduced survival (Garutti et al. 2023; Hébuterne et al. 2014; Liu et al. 2021). Conversely, timely nutrition‐focused management may improve clinical outcomes for patients at nutritional risk, although the effectiveness of such interventions may vary across different clinical settings and patient populations (Kipouros et al. 2023; Muscaritoli et al. 2019; Schuetz et al. 2019).

Although malnutrition has been consistently associated with adverse outcomes in patients with cancer (Kipouros et al. 2023; Liu et al. 2021; Muscaritoli et al. 2019), evidence regarding the clinical benefits of nutritional improvement remains heterogeneous across study populations and care settings (Kaegi‐Braun et al. 2021; Schuetz et al. 2019). Previous multicenter oncology nutrition cohorts in China have provided an important foundation for evaluating nutritional status, quality of life, physical function, nutritional support, medical costs, and survival among patients with common cancers (Xu et al. 2020). However, several limitations remain. Many studies have relied primarily on nutritional information collected at admission, whereas relatively few have examined whether longitudinal changes in nutritional assessment scores are associated with prognosis (Min et al. 2024). Although some studies have incorporated longitudinal nutritional assessments, they often focus on specific types of cancer or treatment contexts (Cho et al. 2022). In addition, the optimal strategy for nutritional screening and assessment in oncology remains uncertain. Different tools may identify different at‐risk populations, and the use of single tool may overlook clinically significant nutritional issues (X. Chen et al. 2023). Evidence from real‐world nutritional support studies is also difficult to interpret because information on nutritional interventions, disease activity, and anticancer treatment is often limited. Consequently, residual confounding may still remain (Kaegi‐Braun et al. 2021).

To address these limitations, the Nutritional Status and Clinical Outcomes in Patients with Common Malignancies (NCOM) multicenter prospective cohort study was established. The NCOM study aims to evaluate nutritional status as a dynamic, multidimensional factor. The study integrates repeated nutritional assessments with clinical outcomes, treatment‐related information, nutritional support, biomarkers, and behavioral and psychosocial factors. This framework enables the study to characterize nutritional trajectories, compare nutritional assessment approaches, and explore modifiable factors associated with nutritional deterioration and subsequent clinical outcomes. This article introduces the cohort framework and presents preliminary baseline characteristics of the NCOM cohort, establishing the foundation for future longitudinal analyses of nutrition‐guided supportive care for patients with common malignancies in Northwest China.

## 2 Methods

### 2.1 Study Design

**FIGURE 1:**
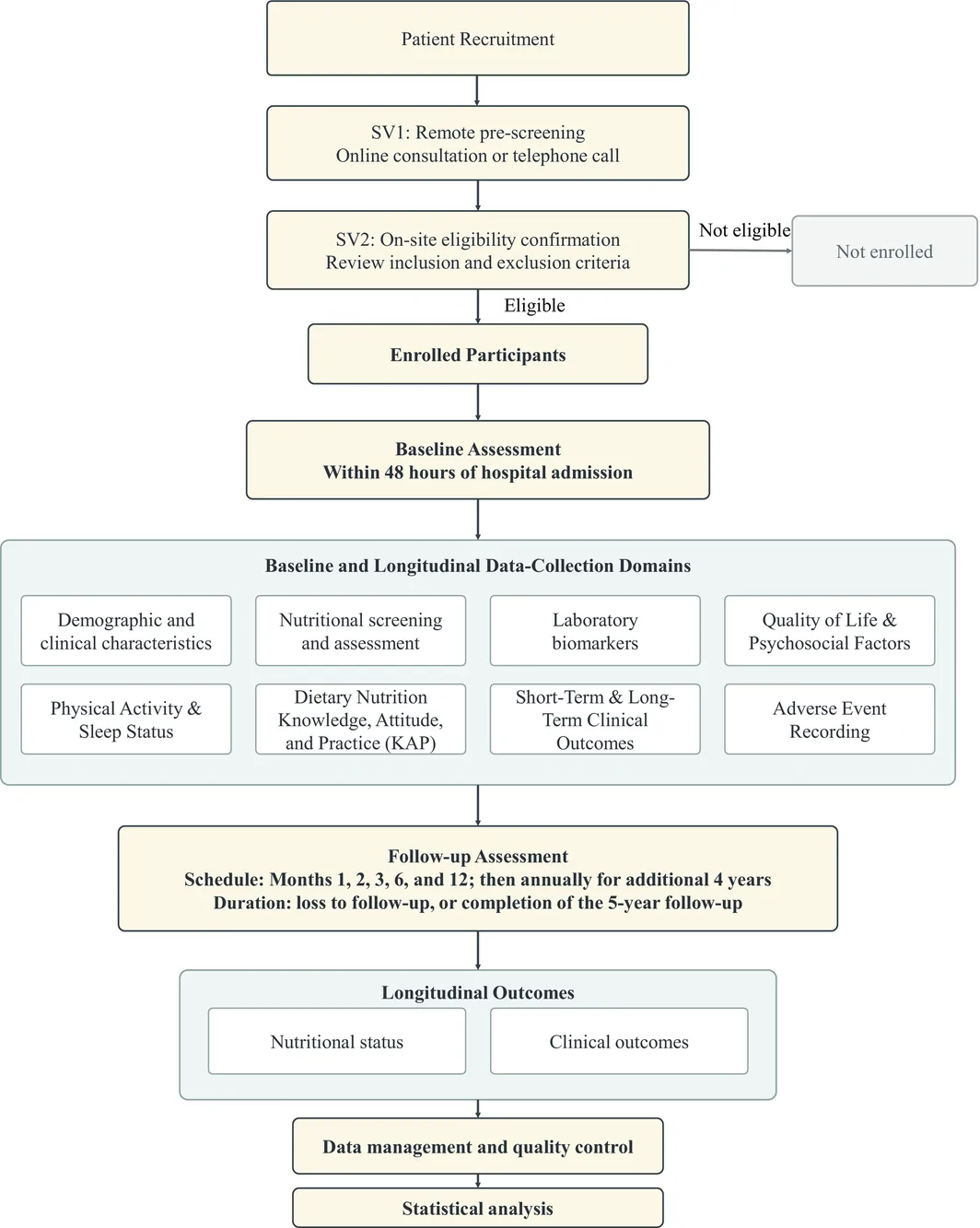
The workflow of the NCOM study.

The NCOM study is an ongoing multicenter, prospective cohort study designed to assess longitudinal changes in nutritional status of patients with cancer and to explore associations between these changes and subsequent clinical outcomes. Participants undergo a baseline assessment within 48 h of hospital admission and are followed at 1, 2, 3, 6, and 12 months, with annual assessments for an additional 4 years or until death or loss to follow‐up (Riboli 2001). Longitudinal assessments include nutritional status, dietary intake, nutritional support, anthropometric measurements, clinical and treatment‐related information, laboratory biomarkers, quality of life, psychosocial factors, physical activity, sleep, and clinical outcomes. The study framework and follow‐up schedule are shown in Figure 1. The study is registered at ClinicalTrials.gov under the identifier NCT06219083.

### 2.2 Study Objectives, Primary Exposure, and Outcome

The primary objectives of the NCOM study are to characterize longitudinal changes in nutritional status and to evaluate the association between baseline nutritional status and subsequent clinical outcomes in patients with cancer. The primary exposure for the analyses is patients' nutritional status. In this study, baseline nutritional status is assessed using the Modified Patient‐Generated Subjective Global Assessment (mPG‐SGA). The mPG‐SGA will be examined using both its total score and three nutritional‐status categories: normal or mild malnutrition (0–2 points), moderate malnutrition (3–6 points), and severe malnutrition (≥ 7 points).

The primary nutritional outcome is longitudinal change in mPG‐SGA during follow‐up, and the primary clinical outcome is overall survival. Secondary outcomes include progression‐free survival, 30‐day clinical events, changes in anthropometric and dietary measures, quality of life, psychological well‐being, sleep quality, and physical activity. Differences across cancer types and treatment modalities, as well as associations between nutritional support and subsequent outcomes, will be examined in secondary or exploratory analyses.

### 2.3 Study Setting and Population

#### 2.3.1 Study Sites

The NCOM study was conducted across 11 tertiary hospitals in Xi'an, Shaanxi Province, China. The participating institutions included Shaanxi Provincial Cancer Hospital; the First Affiliated Hospital of Xi'an Jiaotong University; the Second Affiliated Hospital of Xi'an Jiaotong University; Shaanxi Provincial People's Hospital; the First Affiliated Hospital of the Fourth Military Medical University; the Second Affiliated Hospital of the Fourth Military Medical University; Xi'an Chest Hospital; the Affiliated Hospital of Northwest University Xi'an No. 3 Hospital; Xi'an International Medical Center Hospital; the First Affiliated Hospital of Xi'an Medical University; the Second Affiliated Hospital of Xi'an Medical University.

#### 2.3.2 Recruitment Strategy

Eligible patients will be recruited by clinical staff. Recruitment will primarily occur through direct physician referrals during oncology consultations, supplemented by promotional materials in hospitals, such as posters, brochures, and electronic notices. Patients and their families who learn about the study through these materials can also self‐refer by contacting the research team. The screening process consists of two steps. The first screening visit (SV1) is conducted remotely via an online consultation or phone call to assess basic eligibility, including willingness to participate and key exclusion factors such as pregnancy or concurrent trial participation. Informed consent is obtained at this stage. Patients who pass SV1 will proceed to the second screening visit (SV2), which will be conducted on site at each hospital. During SV2, medical records will be reviewed, and clinical assessments will be performed to confirm whether patients meet the inclusion and exclusion criteria. Only patients who meet all criteria will be formally enrolled.

#### 2.3.3 Inclusion Criteria

- Age ≥ 18 years.
- Histologically confirmed diagnosis of malignancy.
- Patients are scheduled to receive oncologic treatment, including surgery, chemotherapy, radiotherapy, or a combination.
- Mentally competent and able to provide informed consent.
- Willingness to participate in follow‐up assessments.

#### 2.3.4 Exclusion Criteria

Patients will be excluded if they meet any of the following criteria:

- Severe comorbidities that may interfere with participation (e.g., end‐stage liver/kidney disease or severe cardiac failure).
- Patients with HIV/AIDS.
- Prior organ transplantation recipients.
- Expected survival of less than 6 months.
- Pregnant patients.
- Patients currently participating in another interventional clinical trial.

#### 2.3.5 Sample Size

The sample size calculation for the NCOM study was based on 5‐year overall survival (OS). The required number of deaths was calculated using Schoenfeld's event‐based approach and Hsieh and Lavori's extension for a nonbinary covariate (Hsieh and Lavori 2000; Schoenfeld 1983):

$$D=\frac{{\left(Z_{1-\alpha /2}+Z_{1-\beta }\right)}^{2}}{\mathrm{Var}\left(X\right){\left[\ln\left(HR_{\text{step}}\right)\right]}^{2}}$$

*X* denotes the ordered mPG‐SGA category, and $HR_{\text{step}}$ denotes the hazard ratio associated with a one‐category increase in nutritional impairment. For planning purposes, the normal (0) and mild malnutrition (1–2) mPG‐SGA groups were combined, resulting in three ordered categories: normal/mild malnutrition (0–2), moderate malnutrition (3–6), and severe malnutrition (≥ 7). These categories were coded as *X* = 0, 1, and 2, respectively.

Based on the reported group distribution and 5‐year survival data from an independent nationwide multicenter cohort, $\mathrm{Var}\left(X\right)$ and the anticipated event probability were estimated as 0.6873 and 0.6688, respectively (Fu et al. 2022). Previous studies showed progressively poorer OS with worsening mPG‐SGA status and an increased mortality risk associated with mPG‐SGA‐defined malnutrition (Fu et al. 2022; Huo et al. 2023). A planning HR of 1.30 was assumed for the highest versus the lowest category. Under a log‐linear trend across the two category increments, this corresponds to an HR of $1.30^{1/2}=1.1402$ per one‐category deterioration. With a two‐sided α of 0.05% and 80% power, the calculation yielded 663.6 deaths, which was rounded up to 664. This calculation targeted the one‐degree‐of‐freedom log‐linear trend across the ordered mPG‐SGA categories.

A cumulative 35% allowance for loss to follow‐up unrelated to death was adopted as a conservative 5‐year planning assumption, informed by reported attrition in longitudinal oncology cohorts involving repeated multidimensional assessments (Jansen et al. 2022). This loss to follow‐up was assumed to reduce the observable event yield proportionally. The resulting minimum sample size was 1527.4, rounded up to 1528 participants. The recruitment target was set at 1538 participants to provide a small operational margin.

### 2.4 Data Collection

Data collection followed a standardized protocol across all participating hospitals to ensure consistency and accuracy. The baseline assessments will be conducted within 48 h of hospital admission, and well‐trained researchers will collect information on demographic and clinical data, nutritional status, laboratory biomarkers, quality of life, psychosocial factors, physical activity, sleep status, and knowledge, attitudes, and practices (KAPs). Follow‐up assessments will be conducted at predefined intervals to track longitudinal changes in nutritional status and clinical outcomes.

To ensure data reliability, each hospital will designate one or two trained personnel to be responsible for questionnaire administration and clinical data recording. In addition, a supervisor will be appointed to review the integrity of the data and coordinate follow‐up work if necessary. All research personnel must complete standardized training and pass competency assessments to ensure consistency in data collection and minimize variation.

Data management will be centralized through the Research Electronic Data Capture (REDCap) system, which ensures data security, accuracy, and accessibility (Harris et al. 2009). Standardized, validated measurement tools will be used at each site. Data entry will include built‐in validation checks to identify missing values or inconsistencies, with routine audits and cross‐site comparisons performed to maintain high data quality and study validity.

### 2.5 Data Content and Assessment Tools

#### 2.5.1 Demographic and Clinical Data

Upon enrollment, trained personnel will obtain informed consent and record demographic details, including sex, age, marital status, occupation, education, and socioeconomic status. Lifestyle factors such as smoking, alcohol, and tea consumption are documented, given their potential influence on nutritional status and cancer progression (Yuan et al. 2023). Medical history, including chronic conditions, previous diagnoses, medication use, and family history of malignancies, will also be recorded to assess genetic predispositions.

Cancer‐specific data will be collected at baseline and updated during follow‐up to monitor disease progression and treatment response. These data include a pathology‐confirmed diagnosis, TNM staging at first diagnosis, and available molecular markers. The tumor burden will be reassessed at each visit, alongside major treatments (surgery, chemotherapy, radiotherapy, targeted therapy, and immunotherapy), with details on the treatment type, duration, and response. Nutritional support, including enteral or parenteral nutrition in the preceding week, will be tracked to evaluate its impact on treatment outcomes.

##### 2.5.1.1 Karnofsky Performance Status (KPS)

The KPS evaluates a patient's ability to perform daily activities, the presence of physical discomfort, and overall performance status, providing a standardized measure of functional impairment (McNair et al. 2023).

#### 2.5.2 Nutritional Assessments

Nutritional assessments will be conducted via validated screening tools to evaluate nutritional status, risk factors, and dietary intake patterns. Two primary tools and standardized anthropometric measurements will be employed to assess malnutrition risk and severity.

##### 2.5.2.1 mPG‐SGA

The mPG‐SGA scoring system consists of five sections: weight loss history, dietary intake changes, symptoms, activity and function levels, and age. Patients' nutritional status will be categorized on the basis of scores as normal and mild malnutrition (0–2 points), moderate malnutrition (3–6 points), and severe malnutrition (≥ 7 points) (Huo et al. 2023). The version used in this study, mPG‐SGAL11, is an adapted and validated version that was developed for Chinese patients with cancer and incorporates modifications based on nationwide clinical feedback and statistical validation (Fu et al. 2022; Huo et al. 2023).

##### 2.5.2.2 Nutritional Risk Screening (NRS‐2002)

The NRS‐2002 screening tool evaluates nutritional risk on the basis of three components: disease severity, nutritional status, and age. A total score of ≥ 3 indicates nutritional risk, warranting nutritional intervention, whereas a score of < 3 requires weekly reassessment to monitor changes in nutritional status (Zhang et al. 2021).

##### 2.5.2.3 Standardized Anthropometric Measurements

These measurements will include body mass index (BMI), percentage of weight loss, mid‐arm circumference (MAC), triceps skinfold thickness (TSF), and handgrip strength. All measurements will be made under controlled conditions and repeated to ensure accuracy and reliability. To ensure methodological consistency, all measurements will be taken under fasting conditions, with patients wearing minimal clothing and no shoes.

##### 2.5.2.4 Simple Diet Self‐Assessment Tool (SDSAT)

The SDSAT is a brief patient‐reported tool that classifies 1‐day dietary intake into five patterns according to meal consistency and the consumption of staple foods, meat, eggs, milk, oil, and fruit. The patterns are scored from 1 to 5, corresponding to estimated energy intakes of < 300, 300–600, 600–900, 900–1200, and > 1200 kcal/day, respectively. Higher scores indicate greater dietary and estimated energy intake. Three pictorial versions adapted to northern, northwestern, and southern Chinese dietary patterns are available to improve participant understanding (Jin et al. 2020).

##### 2.5.2.5 Nutritional Support in and Out of the Hospital

To assess such support, participants will provide information on the type of nutritional support (enteral or parenteral), formula, and supplemental dose.

#### 2.5.3 Laboratory Biomarkers

Biochemical markers will be analyzed to assess nutritional status, systemic inflammation, and metabolic function. Blood samples will be collected, processed, and stored following standardized protocols to ensure consistency among sites.

Serum ALB, prealbumin, and transferrin will be measured as indicators of protein‐energy malnutrition, providing insights into nutritional depletion and muscle wasting risk. These markers will be analyzed alongside C‐reactive protein (CRP) to account for inflammation‐related reductions in protein levels. Inflammatory cytokines, including interleukin‐6 (IL‐6), interleukin‐1 (IL‐1), and tumor necrosis factor‐alpha (TNF‐α), serve as markers of systemic inflammation and metabolic dysregulation in patients with cancer‐related malnutrition and cachexia (Keller 2019).

Additional biochemical parameters, including hematological markers such as blood glucose, hemoglobin, white blood cell count, neutrophil count, lymphocyte count, red blood cell count, monocyte count, and platelet count, will be recorded where available. Liver and kidney function markers will include AST, ALT, creatinine, blood urea nitrogen, total bilirubin, and direct bilirubin. Lipid‐profile measures will include total cholesterol, triglycerides, high‐density lipoprotein cholesterol (HDL‐C), and low‐density lipoprotein cholesterol (LDL‐C) (Yang et al. 2019).

All values are based on the most recent test results closest to each evaluation. If certain parameters are not routinely available, they will be documented as unavailable. To ensure data reliability, assessments will follow strict quality assurance protocols, including routine calibration of analyzers, standardized sample handling procedures, and interlaboratory validation. When feasible, blood samples will be collected in a fasting state to minimize variability in metabolic markers.

#### 2.5.4 Quality of Life and Psychosocial Factors

A comprehensive assessment of quality of life and emotional well‐being will be conducted to evaluate the psychosocial impact of cancer and its treatment on patients. Standardized and validated measurement tools will be used across study sites to ensure the comparability and reliability of the data.

##### 2.5.4.1 European Organization for Research and Treatment of Cancer Quality of Life Questionnaire (EORTC QLQ‐C30)

This questionnaire is used to evaluate the overall quality of life of patients with cancer. This validated instrument consists of five functional scales (physical, role, cognitive, emotional, and social functions), three symptom scales (fatigue, pain, nausea/vomiting), six single‐item symptom measures (dyspnea, appetite loss, sleep disturbance, constipation, diarrhea, and financial difficulties), and a global quality of life scale. All scales are transformed to scores ranging from 0 to 100. Higher scores indicate better functioning or global health status, whereas higher scores on the symptom and single‐item scales indicate greater symptom burden. This tool has been widely applied in oncology research and provides valuable insights into patients' well‐being, treatment‐related side effects, and overall functionality (Aaronson et al. 1993).

##### 2.5.4.2 Hospital Anxiety and Depression Scale (HADS)

This questionnaire will be used to assess anxiety and depression levels in patients with cancer. This scale consists of two subscales, measuring anxiety (HADS‐A) and depression (HADS‐D), each with seven items. The total score for each subscale ranges from 0 to 21, with the following interpretations: 0–7: normal; 8–10: mild anxiety/depression; 11–14: moderate anxiety/depression; and 15–21: severe anxiety/depression. The HADS is designed specifically for use in hospitalized patients, as it eliminates items related to somatic symptoms that may overlap with cancer‐related physical effects, ensuring a more accurate psychological assessment in this population (Snaith 2003).

#### 2.5.5 Physical Activity and Sleep Status

##### 2.5.5.1 Pittsburgh Sleep Quality Index (PSQI)

This scale consists of seven components (subjective sleep quality, sleep latency, sleep duration, habitual sleep efficiency, sleep disturbances, use of sleeping medications, and daytime dysfunction), with total scores ranging from 0 to 21. A score > 5 indicates poor sleep quality, which is commonly observed in patients with cancer because of disease‐related symptoms, psychological stress, and treatment side effects (Divani et al. 2022).

##### 2.5.5.2 Leisure‐Time Physical Activity Levels

This questionnaire categorizes activity levels into mild, moderate, and vigorous‐intensity exercise. This assessment provides insights into physical activity patterns and their role in cancer‐related fatigue management, functional status, and long‐term patient outcomes. The questionnaire captures the frequency, duration, and intensity of activity to allow for a detailed analysis of the impact of physical activity on patient well‐being (Moore et al. 2016).

#### 2.5.6 Dietary Nutrition Knowledge, Attitude, and Practice (KAP)

The KAP questionnaire is designed to assess nutrition‐related knowledge, attitudes, and practices among patients with cancer. It comprises three components: nutritional knowledge, dietary attitudes, and dietary practices, with a total score ranging from 0 to 104, where higher scores indicate greater nutritional awareness, more positive dietary attitudes, and healthier dietary behaviors (Tang et al. 2023).

The nutritional knowledge section evaluates patients' understanding of nutrient composition, dietary recommendations, and the relationship between nutrition and cancer treatment outcomes. The attitude section measures patients' perceptions and beliefs regarding the importance of nutrition in managing cancer‐related symptoms and treatment side effects. The practice section assesses actual dietary behaviors, including food choices, meal frequency, and adherence to nutritional recommendations.

#### 2.5.7 Short‐Term and Long‐Term Clinical Outcomes

*Short‐term clinical outcomes* will focus on early posthospitalization events within 30 days of admission, providing insights into the immediate impact of nutritional status on patient recovery and complications. These outcomes will include 30‐day survival status, intensive care unit (ICU) admissions, length of stay, and total hospitalization costs, with a specific breakdown of nutrition‐related expenses, such as enteral and parenteral nutrition. Additionally, changes in body weight within 30 days of hospitalization will be monitored as a key indicator of early nutritional deterioration. The incidence of complications, including infections, malnutrition, and drug‐related adverse effects, will be systematically recorded to assess treatment‐related risks. These measures will be used to examine associations between baseline nutritional status and short‐term recovery, treatment complications, and resource utilization.

*Long‐term clinical outcomes* will be assessed through survival and disease‐status tracking at each scheduled follow‐up visit. Overall survival will be defined as the time from baseline assessment to death from any cause. Progression‐free survival will be defined as the time from baseline assessment to the first documented progression or recurrence of the index malignancy, or death from any cause. For patients without an event, overall survival will be censored at the last known date alive, whereas progression‐free survival will be censored at the last adequate disease assessment. At each follow‐up, patients will be classified as alive without documented progression or recurrence, alive with documented progression or recurrence, or deceased, with the cause of death recorded when available.

#### 2.5.8 Adverse Events

Adverse events (AEs) will be systematically recorded and monitored throughout the study to ensure patient safety and data integrity. AEs are defined as any unfavorable or unintended medical occurrence, regardless of their direct relationship with nutritional interventions or treatment. These may include treatment‐related complications, gastrointestinal distress, metabolic imbalances, infections, allergic reactions, or other unexpected health changes.

At each follow‐up visit, trained personnel will actively assess AEs by reviewing medical records and laboratory findings, while participants will be encouraged to self‐report symptoms between visits. Serious adverse events (SAEs), including hospitalization, prolonged disability, or life‐threatening conditions, will be reported following institutional and ethical guidelines.

All AEs and SAEs will be classified by severity, duration, and potential causality, with standardized procedures for data entry, review, and risk assessment. Necessary medical interventions or protocol adjustments will be implemented as needed. This structured monitoring system will support safety monitoring and characterize associations between nutritional support and clinical outcomes.

#### 2.5.9 Follow‐Up Assessments and Data Collection Timeline

Follow‐up assessments will be conducted at predefined intervals to track longitudinal changes in nutritional status, clinical outcomes, and survival outcomes. In the first year, participants will be closely monitored at 1, 2, 3, 6, and 12 months to assess early nutritional changes and treatment‐related complications. Afterward, annual follow‐ups will continue for an additional 4 years or until death or loss to follow‐up, with a focus on survival status and disease progression. Each participant will be enrolled once and assigned a unique participant identifier. Follow‐up time will be calculated from the baseline enrollment date. Data collected during subsequent hospital admissions will be linked to the same participant and assigned to the corresponding follow‐up time point according to the time elapsed since baseline.

In‐person hospital visits will be prioritized for follow‐up to allow comprehensive evaluation of nutritional status, clinical outcomes, and quality of life (Table 1). The questionnaire components required at baseline and each scheduled follow‐up will generally be completed in a single session within 40 min. At baseline and in‐person follow‐up visits, questionnaires will be completed either independently by participants or through researcher‐administered interviews, as appropriate. Assessments will take place in the inpatient ward or a private consultation area under the supervision of trained research personnel. Standardized assistance will be available for participants with reading or physical difficulties, without suggesting or influencing their responses. Participants may pause the assessment or complete it in separate sessions if fatigue or their clinical condition prevents uninterrupted completion.

**TABLE 1:** Schedule of study assessments in the NCOM cohort.

Variable	SV	Follow‐up					
		1st month	2nd month	3rd month	6th month	12th month	Annual follow‐up for an additional 4 years
Demographic and clinical data	✓						
KPS	✓	✓	✓	✓	✓	✓	✓
mPG‐SGA	✓	✓	✓	✓	✓	✓	✓
NRS‐2002	✓	✓	✓	✓	✓	✓	✓
Standardized anthropometric measurements	✓	✓	✓	✓	✓	✓	✓
SDSAT	✓	✓	✓	✓	✓	✓	✓
Nutritional support in and out of hospital	✓	✓	✓	✓	✓	✓	✓
Laboratory biomarkers	✓	✓	✓	✓	✓	✓	✓
EORTC QLQ‐C30	✓	✓	✓	✓	✓	✓	✓
HADS	✓	✓	✓	✓	✓	✓	✓
PSQI	✓	✓	✓	✓	✓	✓	✓
Leisure‐time physical activity levels	✓	✓	✓	✓	✓	✓	✓
KAP Questionnaire	✓				✓	✓	✓
Short‐term clinical outcomes		✓	✓	✓	✓	✓	✓
Long‐term clinical outcomes		✓	✓	✓	✓	✓	✓
AEs	✓	✓	✓	✓	✓	✓	✓

*Note:* Demographic and clinical information is collected at the screening visit. KPS Scoring, mPG‐SGA, NRS‐2002, standardized anthropometric measurements, SDSAT, nutritional support in and out of hospital, laboratory biomarkers, EORTC QLQ‐C30, HADS, PSQI, leisure‐time physical activity levels, and AEs are assessed longitudinally. Follow‐up occurs at 1, 2, 3, 6, and 12 months and annually for an additional 4 years. The KAP questionnaire is administered at screening, 6 months, 12 months, and annual visits. Short‐ and long‐term clinical outcomes are recorded from the first follow‐up onward. Check marks indicate scheduled assessments.

Abbreviations: AEs, adverse events; EORTC QLQ‐C30, European Organization for Research and Treatment of Cancer Quality of Life Questionnaire C30; HADS, Hospital Anxiety and Depression Scale; KAP, Knowledge, Attitude, and Practice questionnaire; KPS, Karnofsky Performance Status; mPG‐SGA, the Modified Patient‐Generated Subjective Global Assessment; NRS‐2002, Nutritional Risk Screening 2002; PSQI, Pittsburgh Sleep Quality Index; SDSAT, Simple Diet Self‐Assessment Tool; SV, screening visit.

When an in‐person visit is not feasible, trained research personnel will conduct the follow‐up by telephone. The telephone assessment will generally be completed within 40 min and will cover the questionnaire components and outcome information specified for that assessment point in Table 1. Measurements requiring physical examination or laboratory testing will not be performed by telephone. The data will instead be obtained from available medical records or collected during a subsequent in‐person hospital visit, as appropriate.

Research personnel will document each follow‐up attempt, including the method of contact, date, and details of the interaction. Participants who miss a scheduled visit will be contacted up to three times by telephone or text message. If contact cannot be established after three attempts, that assessment point will be recorded as a missed visit rather than as an immediate loss to follow‐up. If only some of the scheduled information is obtained, the visit will be recorded as partially completed. All available data will be retained, while uncollected information will be recorded as missing and handled according to the statistical analysis plan. A missed or partially completed visit will not prevent subsequent follow‐up, and contact attempts will continue at later scheduled time points. A participant will be classified as lost to follow‐up only when no further follow‐up information can be obtained despite repeated contact attempts during the remaining follow‐up period. Any known reasons for missed visits, partial completion, withdrawal, relocation, or health deterioration will be documented.

### 2.6 Statistical Analysis Plan

The analyses will follow two principal frameworks. Longitudinal changes in mPG‐SGA will be evaluated using mixed‐effects models. Associations between baseline mPG‐SGA and overall survival will be evaluated using Cox proportional hazards models. Progression‐free survival, short‐term clinical outcomes, other nutritional measures, and patient‐reported outcomes will be examined as secondary outcomes. Additional subgroup, interaction, and nutritional support analyses will be considered exploratory.

Baseline characteristics were summarized as means ± standard deviations (SDs) for normally distributed variables, medians with interquartile ranges (IQRs) for skewed data, and percentages for categorical variables. Continuous variables were compared across the three ordered mPG‐SGA categories—normal or mild malnutrition (0–2 points), moderate malnutrition (3–6 points), and severe malnutrition (≥ 7 points)—using one‐way analysis of variance for normally distributed variables and the Kruskal–Wallis test for non‐normally distributed variables. Categorical variables were compared using the chi‐square test or Fisher's exact test, as appropriate. When the overall test was statistically significant, pairwise comparisons were conducted with appropriate adjustment for multiple comparisons.

Longitudinal changes in nutritional status will be assessed using repeated mPG‐SGA measurements. The mPG‐SGA will be analyzed using both its total score and three‐category classification. Linear mixed‐effects models will be used for total scores when model assumptions are adequately met. Generalized mixed‐effects models with an appropriate link function will be used when those assumptions are not met. Time will initially be modeled as a categorical variable to accommodate potentially nonlinear changes across follow‐up visits. Participant‐specific random intercepts will account for within‐participant correlation, with random slopes considered when supported by the data. Estimated changes from baseline and corresponding 95% confidence intervals will be reported. Ordinal mixed‐effects models will provide complementary analyses of changes among the three malnutrition categories. Generalized estimating equations may be used in sensitivity analyses to estimate population‐average changes. Repeated observations, including those collected during subsequent admissions, will be linked using the unique participant identifier and will not be treated as independent observations. Overall survival will be estimated using the Kaplan–Meier method, with log‐rank tests providing unadjusted comparisons among baseline malnutrition categories. Multivariable Cox proportional hazards models will evaluate associations between baseline mPG‐SGA and overall survival. The mPG‐SGA total score and malnutrition categories will be examined in separate models. Models will adjust for age, sex, cancer type, cancer stage, treatment modality, socioeconomic status, and baseline KPS, subject to the number of observed events. Results will be reported as hazard ratios with 95% confidence intervals. The proportional hazards assumption and the functional form of continuous covariates will be assessed. Stratified models or time‐varying coefficients will be considered if model assumptions are not satisfied. Progression‐free survival will be analyzed as a secondary time‐to‐event outcome using the same general framework.

Secondary short‐term outcomes will be analyzed using models appropriate to their distributions. Multivariable logistic regression will be used for binary outcomes, including 30‐day survival status, ICU admission, infections, and treatment‐related adverse effects. Length of stay, hospitalization costs, and nutrition‐related expenditures will be analyzed using generalized linear models with suitable distributions and link functions. Changes in body weight and other continuous nutritional measures will be analyzed using linear regression or mixed‐effects models, depending on the number of available assessments. Longitudinal quality‐of‐life, psychological, sleep, and physical‐activity measures will primarily be analyzed using mixed‐effects models. Correlation analyses will be considered supplementary and will use Pearson or Spearman coefficients according to variable distributions.

The extent and patterns of missing data will be described for each variable and follow‐up visit. Mixed‐effects models will use all available repeated observations under the missing‐at‐random assumption, without last‐observation‐carried‐forward imputation. Multiple imputation may be applied to missing covariates and selected non‐survival outcomes when the missing‐at‐random assumption is plausible. Complete case analyses will be performed as sensitivity analyses when appropriate. Suspected data errors will be verified, while plausible extreme values will be retained in the primary analyses.

Analyses of nutritional support will be considered secondary observational analyses because treatment allocation may be influenced by baseline malnutrition and disease severity. Propensity score methods may be considered for secondary analyses of nutritional support only when nutritional‐support status, the comparator, eligibility criteria, and time origin can be clearly defined. Subgroup analyses by cancer type, treatment modality, and baseline malnutrition category will be exploratory. Effect modification will be assessed using interaction terms. Secondary and exploratory findings will be reported with effect estimates and 95% confidence intervals, with appropriate caution regarding multiple testing.

By integrating multivariable modeling, survival analysis, longitudinal methods, and approaches to missing data, this study aims to provide robust estimates of associations between nutritional status and clinical outcomes in patients with cancer.

### 2.7 Informed Consent Process

Participation in this study is voluntary, and individuals may withdraw at any time without affecting their medical care. Before enrollment, participants and their families will receive detailed study information, including the study purpose, methodology, potential risks, and benefits. A written informed consent form must be signed, confirming their understanding and voluntary participation.

The informed consent process will be conducted by trained research personnel, ensuring that participants fully understand the study's objectives and procedures; are aware of their right to withdraw at any time without impacting their current or future medical treatment; and can ask questions and receive clarification before signing.

To accommodate diverse literacy levels, consent materials will be provided in clear, accessible language, with verbal explanations when needed. The informed consent form includes both English and Chinese versions.

### 2.8 Data Privacy and Confidentiality

This study strictly adheres to data privacy regulations, ensuring that all participant data remain confidential. Personal identifiers will not be recorded, and all collected data will be deidentified and stored securely in password‐protected electronic databases with restricted access. Data will be used exclusively for research purposes and will not be disclosed or shared beyond the study's scope.

To further protect confidentiality, the following measures will be implemented: data encryption and anonymization before analysis; access to the research database restricted to authorized personnel; and periodic security audits to ensure compliance with institutional and regulatory data protection policies.

### 2.9 Participant Rights and Withdrawal Process

Participants have the right to withdraw at any time without affecting their treatment or medical care. If they choose to withdraw, previously collected data will remain in the research dataset unless they explicitly request removal. In such cases, all identifiable information will be deleted while preserving the scientific integrity of the study.

## 3 Preliminary Baseline Characteristics

As shown in Table 2, 604 patients had been enrolled as of October 2024, and the cohort continues to expand as recruitment proceeds. The mean age was 60.38 ± 11.51 years. Participants aged 60–69 years comprised the largest age group (40.6%), and 64.2% of participants were male.

**TABLE 2:** Preliminary baseline characteristics of participants enrolled in the NCOM cohort through October 2024 stratified by nutritional status according to the mPG‐SGA.

Characteristic	Total (*N* = 604)	Normal/mild malnutrition (*N* = 250)	Moderate malnutrition (*N* = 191)	Severe malnutrition (*N* = 163)
Age (years)	60.38 ± 11.514	58.96 ± 10.983	60.67 ± 11.397	62.20 ± 12.213
**Age category**				
< 45 years	57 (9.4)	26 (10.4)	15 (7.9)	16 (9.8)
45–59 years	186 (30.8)	86 (34.4)	62 (32.5)	38 (23.3)
60–69 years	245 (40.6)	104 (41.6)	77 (40.3)	64 (39.3)
≥ 70 years	116 (19.2)	34 (13.6)	37 (19.4)	45 (27.6)
**Sex**				
Male	388 (64.2)	165 (66.0)	125 (65.4)	98 (60.1)
Female	216 (35.8)	85 (34.0)	66 (34.6)	65 (39.9)
Height (cm)	165.85 ± 7.933	166.68 ± 7.643	165.49 ± 8.097	165.01 ± 8.103
Weight (kg)	63.78 ± 11.909	65.72 ± 11.308	63.62 ± 12.520	60.99 ± 11.570
BMI (kg/m^2^)	23.15 ± 3.830	23.62 ± 3.529	23.15 ± 3.837	22.42 ± 4.163
**Cancer diagnosis**				
Malignant lymphoma	71 (11.8)	42 (16.8)	17 (8.9)	12 (7.4)
Lung cancer	110 (18.2)	52 (20.8)	29 (15.2)	29 (17.8)
Gynecological cancer	24 (4.0)	6 (2.4)	11 (5.8)	7 (4.3)
Colorectal cancer	164 (27.2)	71 (28.4)	55 (28.8)	38 (23.3)
Esophageal cancer	69 (11.4)	14 (5.6)	18 (9.4)	37 (22.7)
Gastric cancer	125 (20.7)	43 (17.2)	51 (26.7)	31 (19.0)
Other tumors	41 (6.8)	22 (8.8)	10 (5.2)	9 (5.5)
**NRS‐2002 assessment**	1.95 ± 1.078	1.48 ± 0.772	1.94 ± 1.067	2.67 ± 1.100
With nutritional risk	141 (23.3)	15 (6.0)	45 (23.6)	81 (49.7)
Without nutritional risk	463 (76.7)	235 (94.0)	146 (76.4)	82 (50.3)
**Economic status**				
No economic hardship	201 (33.3)	88 (35.2)	69 (36.1)	44 (27.0)
Mild economic hardship	332 (55.0)	142 (56.8)	103 (53.9)	87 (53.4)
Moderate economic hardship	54 (8.9)	13 (5.2)	14 (7.3)	27 (16.6)
Severe economic hardship	17 (2.8)	7 (2.8)	5 (2.6)	5 (3.1)
**Residential area**				
Provincial capital city	159 (26.3)	70 (28.0)	53 (27.7)	36 (22.1)
Prefecture‐level city	99 (16.4)	40 (16.0)	33 (17.3)	26 (16.0)
County‐level city	161 (26.7)	71 (28.4)	42 (22.0)	48 (29.4)
Rural area	185 (30.6)	69 (27.6)	63 (33.0)	53 (32.5)
**Educational level**				
No formal education	31 (5.1)	8 (3.2)	10 (5.2)	13 (8.0)
Primary school	134 (22.2)	44 (17.6)	53 (27.7)	37 (22.7)
Secondary school	205 (33.9)	86 (34.4)	62 (32.5)	57 (35.0)
High school	140 (23.2)	66 (26.4)	40 (20.9)	34 (20.9)
Associate degree	55 (9.1)	26 (10.4)	13 (6.8)	16 (9.8)
Bachelor's degree	33 (5.5)	16 (6.4)	12 (6.3)	5 (3.1)
Master's degree or higher	6 (1.0)	4 (1.6)	1 (0.5)	1 (0.6)
**Occupation**				
Farmer	269 (44.5)	96 (38.4)	91 (47.6)	82 (50.3)
Manual worker	24 (4.0)	10 (4.0)	7 (3.7)	7 (4.3)
Employee	83 (13.7)	42 (16.8)	21 (11.0)	20 (12.3)
Retiree	228 (37.7)	102 (40.8)	72 (37.7)	54 (33.1)

*Note:* Characteristics are presented for the overall cohort and according to the three prespecified mPG‐SGA score categories. The table summarizes demographic and anthropometric characteristics, cancer diagnosis, NRS‐2002 results, perceived economic hardship, residential area, educational level, and occupation. Nutritional status categories: normal/mild malnutrition (0–2 points), moderate malnutrition (3–6 points), and severe malnutrition (≥ 7 points). NRS‐2002 nutritional risk: at risk (≥ 3 points) and no risk (< 3 points). Economic status was assessed by patients and their families. No Economic Hardship: No difficulty in covering medical and daily expenses. Mild Economic Hardship: Minor strain, basic needs and expenses can still be met. Moderate Economic Hardship: Significant strain requires adjustments or external support. Severe Economic Hardship: Inability to afford necessary medical or daily expenses without major assistance. Continuous variables are presented as means ± standard deviations (SDs); categorical variables are presented as counts (n) and percentages (%). mPG‐SGA, the Modified Patient‐Generated Subjective Global Assessment; NRS‐2002, Nutritional Risk Screening 2002. These are preliminary baseline data from participants enrolled through October 2024 and will be updated as recruitment continues.

Anthropometric assessments revealed a mean BMI of 23.15 ± 3.83 kg/m^2^, with lower BMI values observed among patients with severe malnutrition (22.42 ± 4.16 kg/m^2^). The cancer diagnosis data revealed that colorectal cancer (27.2%) and gastric cancer (20.7%) were the most prevalent malignancies, followed by lung cancer (18.2%) and malignant lymphoma (11.8%). Notably, esophageal cancer was more prevalent among patients with severe malnutrition. The NRS‐2002 assessment showed that 23.3% of the enrolled patients were at nutritional risk, with a markedly greater proportion in the severe malnutrition group (49.7%).

Socioeconomic factors varied across the cohort, with 55.0% of patients reporting mild economic hardship and 2.8% reporting severe economic hardship. Geographic distribution indicated that 30.6% of patients resided in rural areas, whereas 26.3% lived in provincial capital cities. These disparities underscored the need to evaluate the impact of socioeconomic status on nutritional risk and cancer‐related outcomes. Educational background and occupational data demonstrated further diversity within the cohort: 33.9% of participants had completed secondary school and 44.5% were farmers.

## 4 Discussion

This manuscript presents the cohort framework and preliminary baseline characteristics of the NCOM study, a multicenter prospective cohort established to examine nutritional status and clinical outcomes among patients with common malignancies. Its added value lies not only in documenting the registered study design but also in showing how nutritional impairment can be evaluated within a longitudinal supportive care framework in oncology. The NCOM study integrates longitudinal nutritional assessment with clinical, biochemical, behavioral, and psychosocial information. This approach allows nutritional status to be considered a dynamic, multidimensional factor rather than a single baseline characteristic. Dietary assessment, nutrition‐related biomarkers, and inflammatory indicators may help characterize the biological and clinical context of nutritional deterioration (Phillips et al. 2019; J. Wu et al. 2023). Information on quality of life, psychological distress, and physical activity also provides a basis for examining modifiable factors relevant to supportive care (Y. Wu et al. 2024; Xiao et al. 2019; Zhu et al. 2024). These features support future analyses of how nutritional trajectories may relate to treatment tolerance, adverse events, survival, and patient‐centered outcomes during follow‐up.

The prevalence of malnutrition among patients with cancer varies widely across studies, with reported rates ranging from 20% to more than 70% depending on cancer type, disease stage, treatment setting, and assessment method (Beirer 2021). The preliminary baseline findings of the NCOM study are consistent with this heterogeneity. In the present cohort, nutritional impairment was common, and the distribution of malnutrition differed across tumor types. In particular, esophageal cancer was more prevalent among patients with severe malnutrition. This finding is clinically plausible because upper gastrointestinal tumors may impair swallowing and dietary intake, thereby contributing to nutritional deterioration during treatment. Previous evidence has likewise shown that malnutrition risk varies substantially by cancer site among patients with solid tumors at diagnosis (Kadakia et al. 2022).

Socioeconomic characteristics may further shape nutritional vulnerability in this cohort. A considerable proportion of participants reported economic hardship or lived in rural areas. Participants' educational and occupational backgrounds may also affect dietary resources, access to nutritional support, and continuity of care. Although the current baseline data cannot establish causal relationships, the findings are consistent with previous evidence linking lower socioeconomic status to a higher risk of malnutrition (Sandström et al. 2023). This association may reflect differences in living conditions, nutrition, and access to healthcare. Therefore, the NCOM cohort provides an opportunity to examine nutritional risk not only as a tumor‐ or treatment‐related problem but also as a clinical issue embedded in broader social and healthcare contexts. Previous intervention studies suggest that nutrition‐focused strategies may improve selected nutritional and treatment‐related outcomes in specific oncology settings. However, their effects may vary by population, treatment context, and intervention type (Li et al. 2024).

The baseline comparison of nutritional assessment tools is an important feature of the NCOM study. In this cohort, 58.61% of patients were classified as having moderate or severe malnutrition according to the mPG‐SGA, whereas 23.34% were identified as being at nutritional risk by the NRS‐2002. This difference should not be interpreted simply as disagreement between the tools. Rather, it may reflect differences in their purposes, scoring structures, and clinical emphases. PG‐SGA‐based assessments are widely used in oncology because they capture multiple nutrition‐related domains, including weight change, dietary intake, nutrition‐impact symptoms, and functional status (H.‐J. Zhou et al. 2021). The lower proportion identified by the NRS‐2002 suggests that rapid screening tools may identify a narrower group of patients requiring immediate nutritional attention. In contrast, comprehensive assessment tools may detect a broader spectrum of nutritional impairment. These findings are consistent with previous evidence suggesting that the PG‐SGA may be more sensitive than the NRS‐2002 for identifying nutrition‐related problems in patients with cancer (X. Chen et al. 2023). They also support selecting screening and assessment strategies according to their intended clinical purpose.

The clinical significance of the NCOM cohort lies in its potential to clarify how changes in nutritional status relate to treatment tolerance, adverse events, and survival during follow‐up. Previous studies have shown that malnutrition is associated with poorer treatment tolerance and a higher risk of treatment‐related complications, including gastrointestinal toxicity and myelosuppression (Li et al. 2024). These complications may contribute to dose reductions, treatment delays, and poorer clinical outcomes. In the authors' previous study of patients with non‐Hodgkin lymphoma, deterioration in nutritional status during chemotherapy was associated with chemotherapy‐related toxicities (Q. Zhou et al. 2025). This finding supports the importance of monitoring nutritional status throughout treatment rather than relying solely on baseline assessment. Therefore, the repeated follow‐up design of the NCOM study may help identify patients vulnerable to nutritional decline and provide evidence to inform timely nutrition‐focused supportive care.

This study has several strengths. The prospective multicenter design and planned repeated longitudinal follow‐up assessments provide a framework for evaluating nutritional status as a longitudinal exposure. The study integrates validated nutritional screening and assessment tools with anthropometric, biochemical, dietary, clinical, and psychosocial information. This integration will enable future analyses of nutritional trajectories and their associations with treatment‐related and survival outcomes. In addition, the preliminary baseline data presented in this manuscript demonstrate the nutritional, clinical, and socioeconomic heterogeneity of the enrolled population. These findings support the importance of continued follow‐up in this cohort.

Several limitations should also be acknowledged. First, the present manuscript primarily reports the study framework and preliminary baseline characteristics. Therefore, the present baseline data cannot establish associations between nutritional status and clinical outcomes or support causal inferences. The preliminary baseline data also reflect recruitment only through October 2024. The cancer‐type distribution may have been influenced by participating clinical departments, referral patterns, and the early recruitment phase, potentially underrepresenting some common malignancies. As the cohort expands, future analyses will report updated cancer‐type distributions and examine nutritional status across a broader range of malignancies. Second, dietary intake and some behavioral variables are self‐reported and may be affected by recall or reporting bias. Objective indicators, including anthropometric measurements and biochemical markers, may partly complement the self‐reported information collected in this study. Third, loss to follow‐up may occur because of disease progression, death, relocation, or withdrawal, although the sample size calculation accounted for potential attrition (Reeves et al. 2007; Schwedhelm et al. 2016). Fourth, the cohort is currently limited to hospitals in Xi'an, which may restrict generalizability of the findings to other regions. Nevertheless, as a major regional referral center in Northwest China, Xi'an may attract patients from diverse geographic and socioeconomic backgrounds (N. Chen et al. 2022). Finally, because this is an observational cohort, residual confounding cannot be fully excluded, even with multivariable adjustment and longitudinal modeling. The study also does not include in‐depth analyses of molecular or metabolic pathways. This limitation restricts its ability to directly investigate the biological mechanisms linking malnutrition to cancer progression. Future studies incorporating mechanistic biomarkers may help address this issue.

## 5 Conclusion

The NCOM study establishes a multicenter prospective cohort framework for evaluating longitudinal nutritional status and clinical outcomes among patients with common malignancies. Preliminary baseline findings indicate a substantial burden of nutritional impairment and differences between nutritional assessment approaches. These findings support the need for early identification and repeated longitudinal nutritional monitoring in oncology care. Continued recruitment and follow‐up are needed to determine how nutritional trajectories are associated with treatment tolerance, adverse events, quality of life, and survival. These analyses may provide evidence to inform nutrition‐focused supportive care and future intervention research.

## Author Contributions

**Hexiang Yang:** conceptualization, methodology, software, data curation, investigation, validation, formal analysis, supervision, visualization, project administration, writing – original draft, writing – review and editing, resources. **Yaxin Yu:** investigation, conceptualization, validation, formal analysis, software, project administration, writing – original draft. **Haige Cao:** visualization, formal analysis. **Feng Liu:** formal analysis, visualization. **Jiahe Zhou:** investigation, software. **Yuluyuan Tian:** methodology, investigation. **Yilan Li:** software, validation. **Xinyue Wang:** data curation, validation. **Yushuo Sun:** methodology, data curation. **Xiaoqin Luo:** funding acquisition, writing – review and editing, validation, investigation, supervision, resources.

## Funding

This work was funded by the Natural Science Basic Research Program of Shaanxi Province (Program No. 2020JQ‐460) and the CNS‐Nutrilite Science Popularization Fund for Rational Diet & Nutritious Breakfast Pattern (CNS‐RDSF‐2025‐001).

## Ethics Statement

All methods and procedures were conducted in accordance with relevant guidelines and regulations, in line with the principles of the Declaration of Helsinki. Ethical approval for this study was obtained from the Institutional Review Board of Xi'an Jiaotong University (Approval No. 2022‐1373).

## Consent

All participants were fully informed of the purpose and potential benefits of the study, and written informed consent was obtained prior to participation. Confidentiality of participant information was strictly maintained throughout the study.

## Conflicts of Interest

The authors declare no conflicts of interest.

## Data Availability

The data that support the findings of this study are available from the corresponding author upon reasonable request.
